# Clinical Outcomes after Endovascular Revascularisation of the Femoropopliteal Arterial Segment in Patients with Anticoagulant versus Antiplatelet Therapy: A Single-Centre Retrospective Cohort Study

**DOI:** 10.3390/jcdd9070207

**Published:** 2022-06-30

**Authors:** Kevin Pelicon, Klemen Petek, Anja Boc, Vinko Boc, Nataša Kejžar, Tjaša Vižintin Cuderman, Aleš Blinc

**Affiliations:** 1 Department of Vascular Diseases, University Medical Centre Ljubljana, 1000 Ljubljana, Slovenia; kevin.pelicon@mf.uni-lj.si (K.P.); klemen.petek@mf.uni-lj.si (K.P.); anja.boc@mf.uni-lj.si (A.B.); vinko.boc@kclj.si (V.B.); tjasa.vizintin@kclj.si (T.V.C.); 2 Institute of Anatomy, Faculty of Medicine, University of Ljubljana, 1000 Ljubljana, Slovenia; 3 Institute for Biostatistics and Medical Informatics, Faculty of Medicine, University of Ljubljana, 1000 Ljubljana, Slovenia; natasa.kejzar@mf.uni-lj.si; 4 Department of Internal Medicine, Faculty of Medicine, University of Ljubljana, 1000 Ljubljana, Slovenia

**Keywords:** peripheral arterial disease, endovascular procedures, femoral artery, anticoagulants, platelet aggregation inhibitors, effectiveness, safety, propensity score, treatment outcome

## Abstract

To prevent atherothrombotic events, patients with peripheral arterial disease are typically prescribed antiplatelet therapy (APT). However, some of them receive anticoagulant therapy (ACT) due to comorbidities. Our aim was to determine possible differences in the effectiveness and safety of both treatments in patients after endovascular femoropopliteal revascularisation. We retrospectively analysed 1247 patients after successful femoropopliteal revascularisation performed in a single tertiary medical centre and classified them into the ACT or APT group, based on their prescribed treatment. The groups were characterised by descriptive statistics, and their characteristics were adjusted for confounders by propensity score matching. Effectiveness and safety outcomes were assessed within one year after revascularisation. The odds ratio for the composite outcome of all-cause death, PAD exacerbation, and major amputation due to vascular causes with ACT versus APT was 1.21 (95% CI 0.53–2.21; *p* = 0.484). The odds ratio for major bleeding as defined by the International Society on Thrombosis and Haemostasis with ACT versus APT was 0.77 (95% CI 0.13–3.84; *p* = 0.251). We found no statistically significant difference in the effectiveness and safety of ACT, when compared to APT in patients with similar cardiovascular risk factors and other baseline characteristics. Further prospective research is warranted.

## 1. Introduction

Patients with peripheral arterial disease (PAD) are at an increased risk of both major adverse cardiovascular events (MACEs) and major adverse limb events (MALEs) [1,2,3]. Antithrombotic therapy thus represents a principal treatment modality, with patients generally being prescribed antiplatelet therapy (APT) [4]. However, recent studies suggest that the addition of low-dose anticoagulant therapy (ACT) to APT in patients with stable PAD yields superior outcomes compared to APT alone [5]. Revascularisation procedures pose additional risk for ischaemic events, with MALEs occurring mainly in the first year after the procedure and MACEs staying stably elevated in the long-term [6]. Post-procedural antithrombotic therapy with the combination of APT and low-dose ACT can improve outcomes after revascularisation [7].

Due to comorbidities, such as atrial fibrillation, artificial heart valves, or a history of venous thromboembolism, some PAD patients receive full-dose ACT [1]. Despite its widespread use, the effectiveness of full-dose ACT in preventing MACEs and MALEs in patients with PAD remains unclear. Long-term patency is especially poor after revascularisation of the femoropopliteal arterial segment [8,9]. Further research into the optimal post-procedural antithrombotic regimen, aimed at improving outcomes and prolonging patency, is thus crucial. In this retrospective cohort study, we used propensity score matching (PSM) to compare the effectiveness and safety outcomes within one year after femoropopliteal revascularisation between patients who were prescribed full-dose ACT with or without the transient addition of APT and patients who received APT alone.

## 2. Materials and Methods

We retrospectively analysed the cohort of PAD patients with disabling intermittent claudication or chronic critical limb ischaemia, who underwent successful femoropopliteal endovascular revascularisation in the Catheter Laboratory of the Clinical Department of Vascular Diseases of the University Medical Centre Ljubljana, Slovenia, between 2014 and 2019. The exclusion criteria were insufficient data on post-procedural antithrombotic treatment, a simultaneously performed surgical bypass procedure, and the presence of previously undiagnosed iliac lesions that required another intervention, despite patency of the originally treated femoropopliteal segment. If the same patient underwent multiple revascularisation procedures between the years 2014 and 2019, only the first successful procedure was considered. When revascularisation was performed simultaneously on both limbs, the limb with more severe symptoms was considered. Part of the patient population was previously studied in a retrospective study examining the effectiveness and safety of ACT and APT after endovascular revascularisation of all lower-limb arterial segments [10].

Patients were treated according to the ESC guidelines [11], with bailout stenting performed in case of significant residual stenosis, early elastic recoil, or flow-limiting arterial wall dissection. As a rule, patients were already receiving APT before the procedure, while those without it received 100 mg of acetylsalicylic acid a day before and on the day of the procedure. During the revascularisation procedure, all patients received 3000 to 5000 I.U. of unfractionated heparin intravenously. After the procedure, most patients were prescribed APT. In case of stent placement or the use of a drug-coated device, as well as in the majority of patients with simultaneous angioplasty of the popliteal or below-knee arteries, dual APT (DAPT) was prescribed, most commonly for 3 months. Patients with comorbidities which called for ACT were prescribed therapeutic doses of ACT, sometimes along with transient APT, usually for 1 to 3 months, depending on the interventionists’ assessment and the patient’s risk of bleeding.

We collected data on patient demographics, cardiovascular risk factors, markers of PAD severity, previous revascularisation procedures and amputations, procedural characteristics, and prescribed pre- and post-procedural medications. The presence of cardiovascular risk factors and comorbidities was determined based on established diagnoses, available laboratory analyses, and prescriptions of specific medication. PAD severity was assessed clinically using the Fontaine classification [12], and the extent of lesions was evaluated using the TASC II scoring system [2]. Run-off was assessed using modified criteria of the Society for Vascular Surgery [13] and deemed good in patients with a patent popliteal and at least two below-knee arteries. The patients’ data were retrieved from the hospital’s electronic medical records and further supplemented with information obtained from the patients’ general practitioners if necessary. Data were matched by the hospitalisation number identifier and anonymised for analysis.

Two treatment groups were formed based on the patients’ prescribed antithrombotic therapy upon discharge from the hospital. Patients who received only APT were in the APT group, while patients who received full-dose ACT with or without transient APT were in the ACT group. Possible subsequent changes of antithrombotic therapy during the observed interval and poor patient adherence were not considered. 

**Figure 1:**
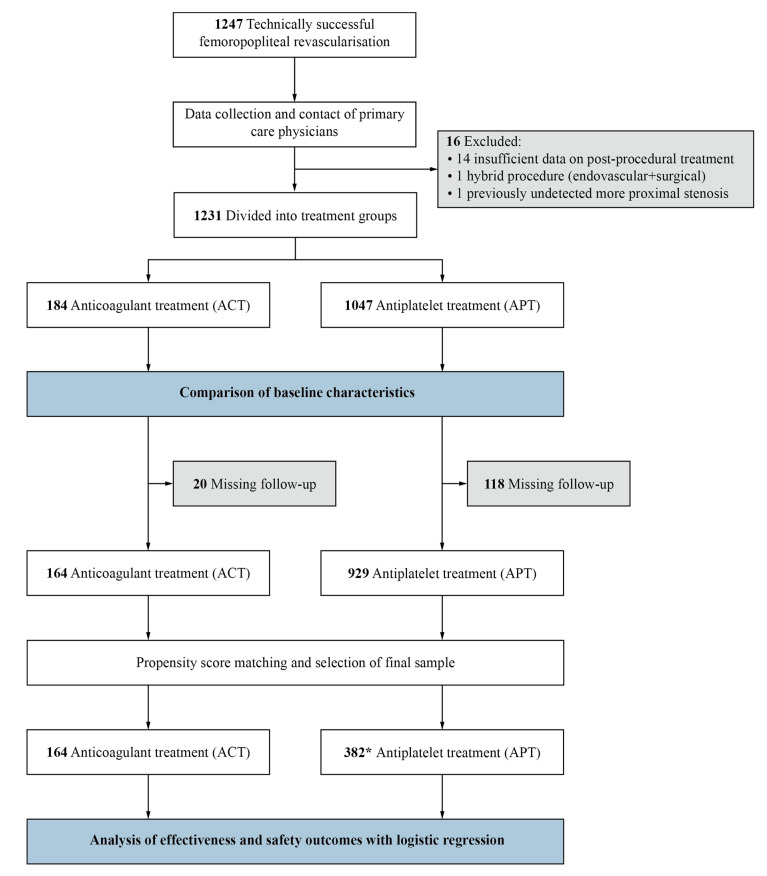
The studied patient profile, process of exclusion, and analysis. * is the number of matched patients, not considering the patients’ statistical weights. The effective sample size was 243.1 patients.

We analysed all patients’ medical records within one year after the first successful procedure, or until the occurrence of an adverse event included in the composite effectiveness outcome if it occurred sooner. In patients without follow-up examinations within one year after the procedure, we assessed the outcomes based on later follow-up examinations when available or gathered information from the patients’ general practitioners. The primary aim of the study was the comparison of the effectiveness and safety outcomes between the ACT and the APT groups within one year after the procedure, and the secondary aim was the comparison of the two groups in terms of their baseline characteristics. Patients for whom both outcomes one year after the procedure could not be assessed were included only in the comparison of baseline characteristics, and not in the PSM procedure and comparison of effectiveness and safety outcomes (Figure 1).

The effectiveness outcome was defined as a composite of the following adverse events: all-cause death, exacerbation of PAD symptoms, and previously unplanned major amputation of the treated limb due to vascular causes. The safety outcome was defined as the occurrence of major bleeding as defined by the International Society on Thrombosis and Haemostasis (ISTH) [14].

The patients’ baseline characteristics were analysed using the SPSS statistical package (version 25, IBM SPSS Statistics, Armonk, NY, USA). Categorical variables were reported as frequency and percentage, and continuous variables as mean ± standard deviation (SD). Comparisons of the studied groups were performed using Pearson’s χ^2^ or Fisher’s exact tests for categorical variables and independent sample *t*-tests for continuous variables. All tests were two-tailed and used a significance level of *p* ≤ 0.05 for descriptive purposes.

PSM was used to account for confounding factors and create comparable risk groups. The patients’ baseline characteristics were used as covariates to calculate the propensity scores. Standardised mean differences (SMDs) were calculated for all covariates, with balance defined as SMD < 0.05. We attempted multiple matching algorithms (nearest neighbour, optimal, full), with the 1:5 nearest neighbour with replacement algorithm yielding the most adequate balance, meaning the fewest covariates with SMD > 0.05. Matching with replacement meant every patient in the APT group could be assigned to multiple patients in the ACT group. Patients in the APT group were thus given statistical weights, signifying their importance in the final sample. The final sample included all patients in the ACT group and only their matched counterparts in the APT group, considering their weights. The relative treatment effect was assessed by fitting a logistic regression model to the matched sample with the patients’ treatment group, propensity scores, and atrial fibrillation (not included when calculating propensity scores) as predictors and either effectiveness or safety as the outcome. Confidence intervals were produced with g-computation by bootstrapping [15]. Two subanalyses were performed, considering the separate components of the composite effectiveness outcome (all-cause death and MALEs), as well as two sensitivity analyses of treatment effects—one using multiple logistic regression (on an unmatched sample, i.e., without PSM) and another using the same methodology as the main analysis, assuming the best-case scenario (favourable effectiveness outcomes) for the 138 patients with missing follow-ups; see Supplementary Materials. PSM and logistic regression were performed in the R programming language [16,17].

## 3. Results

In the six-year period between January 2014 and December 2019, successful femoropopliteal revascularisation was performed in 1247 patients, 1231 of whom were included in our analysis. Of these patients, 14.9% (184 of 1231) were prescribed ACT. The baseline characteristics of the ACT and APT groups are shown in Table 1.

The prescribed post-procedural treatment regimen is shown in Table 2. In the ACT group, the most prescribed anticoagulant was warfarin (134 of 184 patients; 72.8%), followed by rivaroxaban (17 of 184 patients; 9.2%), and dabigatran and apixaban (each prescribed to 13 of 184 patients; 7.1%). Treatment with low-molecular-weight heparin and acenocoumarol was rare, with only 5 and 2 patients receiving these anticoagulants, respectively.

Not all patients completed follow-ups, meaning the effectiveness and safety outcomes could not be determined for 138 patients. After excluding them from further analysis, 1093 patients remained, 164 (15%) of which were in the ACT group (Figure 1). Table 3 shows the effectiveness and safety outcomes for both groups before performing PSM. The unmatched groups were not compared with respect to the effectiveness and safety outcomes as the groups’ baseline characteristics differed fundamentally, giving little value to any comparison prior to PSM.

Of the 21 major bleeding events that were noted in the observed period, 5 (23.8%) occurred at the access site shortly after the procedure, and 10 (47.6%) occurred while the patients were prescribed multiple antithrombotic agents (either the combination of ACT and APT or DAPT). 

**Figure 2:**
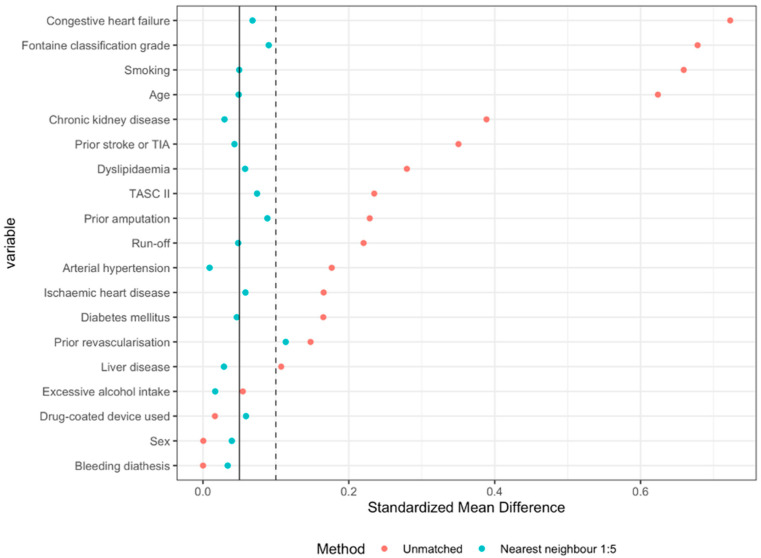
Graphic presentation of the dispersion of standardised mean difference (SMD) values for the group of patients treated with full-dose anticoagulant therapy and the group of patients treated with antiplatelet therapy, before and after matching with the nearest-neighbour 1:5 algorithm with replacement.

After PSM, the sample size consisted of all 164 patients in the ACT group and 382 patients in the APT group who matched best with the patients in the ACT group. When accounting for their statistical weights, the effective sample size of the APT group was 243.1 patients. The final SMD values for all covariates before and after matching are listed in Table 4 and shown graphically in Figure 2. Matching yielded adequate balance.

After matching, we found no statistically significant difference in the effectiveness or safety of either treatment regimen. The odds ratio (OR) for the adverse effectiveness outcome in ACT versus APT was 1.21 (95% CI 0.53–2.21; *p* = 0.484). In the subanalysis, the OR for all-cause death was 0.56 (95% CI 0.24–1.29; *p* = 0.176), and the OR for MALEs was 1.61 (95% CI 0.77–3.37; *p* = 0.204). 

The OR for major bleeding with ACT versus APT was 0.77 (95% CI 0.13–3.84; *p* = 0.251). 

The results of the sensitivity analyses concur with the reported findings, i.e., statistically non-significant effects (see Supplementary Materials).

## 4. Discussion

While the long-term patency after endovascular revascularisation of the iliac arteries is more than 70% [18], the 5-year reocclusion rates of the femoropopliteal segment exceed 50% [8]. One of the main challenges of PAD treatment therefore lies in improving the long-term outcomes after femoropopliteal endovascular revascularisation, either through advances in the procedural techniques or by adjusting the peri-procedurally prescribed medication. Bare-metal stent placement, which is effective in the iliac segment, does not adequately increase the long-term femoropopliteal patency rates [19]. Drug-eluting devices seem to offer an effective alternative [8]; however, their use is associated with considerable expense and, according to some studies, with increased late all-cause death [20] and limb amputations [21]. As for the medication regime, the optimisation of post-procedural antithrombotic therapy could be one of the ways to improve procedural outcomes, since the revascularisation procedure itself poses an elevated risk of atherothrombotic events by damaging the vessel’s wall, stimulating platelet adhesion, and causing local inflammation [1,6,11,22,23,24,25]. Unfortunately, high-level evidence on the optimal antithrombotic treatment in PAD patients after revascularisation remains scarce, and guidelines are extrapolated from the field of percutaneous coronary interventions to a large degree. At present, the most promising regimen appears to be a combination of low-dose ACT and APT [26]. Nevertheless, some patients will continue to require full-dose ACT due to their comorbidities. Understanding its effectiveness and safety compared to the more commonly prescribed APT will thus continue to be of importance. 

Atrial fibrillation is one of the most common indications for anticoagulant use [27]; therefore, its presence in almost 80% of our patients in the ACT group was not surprising. Patients with atrial fibrillation are usually older, have more generalised atherosclerosis and more associated illnesses, making their outcomes less favourable [28]. Furthermore, atrial fibrillation is a strong predictor of MACEs and all-cause death in patients with symptomatic PAD [29]. These facts are in line with our findings. Compared to patients in the APT group, patients in the ACT group were almost 6 years older and had a higher prevalence of almost all observed cardiovascular risk factors, including arterial hypertension, diabetes mellitus, chronic kidney disease, and ischaemic heart or cerebrovascular disease. Furthermore, these patients had more advanced PAD, with chronic critical limb ischaemia and prior amputations being about twice as common in the ACT group compared to the APT group. In the ACT group, pre-procedural values of ABI > 1.4, indicating medial arterial calcification, were measured in 14% of patients, compared to only 5% in the APT group. This could be explained by the higher prevalence of diabetes and chronic kidney disease in the ACT group, which are both risk factors for the development of medial arterial calcification [2,6]. Interestingly, there was no difference in the complexity of the lesions as defined by the TASC II classification. 

To compare the outcomes of our two groups of patients, we used PSM. This method is being increasingly used in observational studies, as it allows for the inclusion of a wide range of covariates and thus enables the comparison of groups of patients with radically different characteristics. To calculate propensity scores, we used various markers of PAD severity, cardiovascular risk factors, and other patient-specific covariates. As patients in the APT group were matched to patients in the ACT group, the final sample had characteristics which were similar to those of the ACT group, namely older patients, those with more advanced PAD, and those with more comorbidities. Notably, atrial fibrillation was not included as a covariate as it predicted the patients’ group with near certainty. 

Among the propensity-matched groups, we found no statistically significant difference in the cumulative incidence of all-cause death, PAD exacerbation, or previously unplanned major amputation due to vascular causes. However, the calculated confidence interval was wide, indicating the need for further, possibly prospective, research. This is especially true when considering the fact that antithrombotic treatment after revascularisation is only one of a multitude of factors which affects a revascularisation procedure’s outcomes. 

Recent studies show that the addition of low-dose rivaroxaban to acetylsalicylic acid reduces the incidence of MACEs and MALEs compared to acetylsalicylic acid alone, both in stable PAD [5] and after revascularisation [26]. However, similar to our findings, research examining full-dose ACT after percutaneous infra-inguinal revascularisation has failed to show a statistically significant difference between patients who received ACT and APT, meaning that the addition of full-dose ACT did not correlate with improved arterial patency [30,31]. Importantly, most comparable studies examining the effects of antithrombotic treatment after revascularisation consider specific antithrombotic agents, while our patients were divided into the ACT or APT group based only on the general type of antithrombotic treatment. For patients who require full-dose ACT due to comorbidity and have a low bleeding risk, current treatment guidelines recommend the use of dual-antithrombotic therapy with an anticoagulant drug in combination with either acetylsalicylic acid or clopidogrel for as limited a duration as possible (1 month), depending on the clinical indication and bleeding risk [11]. According to clinical guidelines, about half of our patients in the ACT group therefore temporarily received APT, which may have influenced the outcome to some extent. 

Major bleeding occurred in 3.6% of our patients in the unmatched ACT group and was fatal for 0.6%. These results are within the reported ranges, as about 2–5% of patients undergoing ACT are expected to experience major bleeding each year [32], while fatal bleeding is expected in up to 0.5% of patients [33]. By contrast, the incidence of major bleeding in our unmatched APT group was 1.6%, with 0.2% fatal bleedings. The annual risk of major bleeding with single-antiplatelet therapy is 1–1.5% [34], but more than half of the patients in our APT group received DAPT in the first months after revascularisation. As DAPT has been shown to increase bleeding risk 2- to 3-fold [35], this makes for a possible explanation for the higher-than-expected incidence of major bleeding. It is important to note that, excluding bleeding events which were a direct result of the procedures, almost half of the recorded major bleeding events occurred within the first three months after revascularisation. This was the period when most patients were prescribed more than one antithrombotic agent—either ACT with APT or DAPT. After multivariate regression analysis of the propensity-matched groups, we found no statistically significant difference in the occurrence of the safety outcome, i.e., major bleeding as defined by the ISTH; however, the confidence interval was wide. Prior studies indicate a higher risk of bleeding complications in ACT compared to APT alone [30]. The lack of difference between our balanced study groups can partially be explained by the expectedly low number of major bleeding events. Furthermore, the probability of major bleeding depends not only on the type of antithrombotic drug used, but also on the patients’ risk for bleeding. After matching the younger and healthier APT group to the older and more morbid ACT group, both groups had a considerable bleeding risk.

An important limitation of our study was its retrospective nature, which did not enable us to consider factors such as poor adherence to prescribed treatment or changes in antithrombotic therapy. For some patients, neither composite outcome could be assessed. As it was unclear if these patients had completed follow-ups due to favourable clinical outcomes or other causes, such as being treated at a different institution, they were excluded before PSM. Follow-up information (in the form of a clinical examination, repeated intervention, amputation, death) was, however, available for almost 90% of our patients, making selection bias unlikely [36]. Despite this, the final effective sample size after PSM was relatively small, especially considering the safety outcome, since major haemorrhages were a rare event. Minor haemorrhages have a potentially significant effect on a patient’s quality of life; however, we were unable to assess their frequency as, by definition, they do not always require medical treatment [14,37]. Hence, minor bleeding was not routinely recorded in the patients’ documentation and was therefore excluded from analysis to prevent bias. In addition to the abovementioned points, when drafting potential future (prospective) research, the use of modern imaging techniques such as CT perfusion imaging of the lower limb should be considered to assess treatment outcomes. In our study, outcomes were only assessed clinically, while recent research shows non-invasive imaging to be a viable alternative [38].

## 5. Conclusions

Among PAD patients requiring percutaneous femoropopliteal revascularisation, those treated with full-dose ACT were significantly older, had more advanced PAD, and expressed a higher prevalence of most cardiovascular risk factors. After matching the ACT and APT groups, we found no statistically significant difference in either group’s composite effectiveness outcome of all-cause death, exacerbation of PAD symptoms, or previously unplanned major amputation of the treated limb due to vascular causes. Furthermore, we found no statistically significant difference in the safety outcome, defined as a major bleeding event. However, the width of our results’ confidence intervals prevents us from drawing definitive conclusions on the equivalence of both treatment regimes in terms of their effectiveness and safety. As full-dose ACT will continue to be used in PAD patients with some comorbidities who need a revascularisation procedure, additional research is warranted to clarify the effectiveness and safety of ACT in the post-procedural period. This is of special relevance, considering that the recommended short-term addition of APT to ACT further elevates the risk of bleeding in this already frail group of patients.

## Figures and Tables

**Table 1:** Baseline characteristics of the group of patients treated with full-dose anticoagulant therapy and the group of patients treated with antiplatelet therapy.

	Anticoagulant Group N = 184	Antiplatelet Group N = 1047	*p* Value
**Patient Demographics**			
Age (years)	76.4 ± 10.5	70.5 ± 10.3	**<0.001**
Female sex	77 (41.8)	459 (43.8)	0.615
**Markers of PAD severity and procedural characteristics**			
Fontaine classification grade			**<0.001**
2b	76 (41.3)	743 (71.0)	
3	25 (13.6)	69 (6.6)	
4	83 (45.1)	235 (22.4)	
Pre-procedural ABI			**<0.001**
>1.40	26 (14.1)	55 (5.3)	
0.91–1.40	15 (8.2)	57 (5.4)	
0.40–0.90	86 (46.7)	680 (65.0)	
<0.40	32 (17.4)	129 (12.3)	
TASC II			0.157
A	26 (14.1)	187 (17.9)	
B	81 (44.0)	443 (42.3)	
C	65 (35.3)	379 (36.2)	
D	12 (6.5)	36 (3.4)	
not assessed	0 (0.0)	2 (0.2)	
Drug-coated device used *	5 (2.7)	35 (3.3)	0.659
Prior revascularisation			0.222
of the same segment	36 (19.6)	169 (16.1)	
of a different segment	19 (10.3)	151 (14.4)	
Prior amputation			**0.008**
below the ankle	16 (8.7)	56 (5.3)	
above the ankle	9 (4.9)	20 (1.9)	
Poor run-off (percentage of assessed) ^†^	55/140 (39.3)	256/829 (30.9)	**0.049**
**Cardiovascular risk factors and associated illnesses**			
Arterial hypertension	170 (92.4)	909 (86.8)	**0.034**
Dyslipidaemia, treated with statins	128 (69.6)	821 (78.4)	**0.008**
Diabetes mellitus	94 (51.1)	450 (43.0)	**0.041**
Atrial fibrillation	145 (78.8)	37 (3.5)	**<0.001**
Ischaemic heart disease	58 (31.5)	229 (21.9)	**0.004**
Congestive heart failure	73 (39.7)	114 (10.9)	**<0.001**
Prior stroke or TIA	44 (23.9)	120 (11.5)	**<0.001**
Chronic kidney disease	77 (41.8)	267 (25.5)	**<0.001**
Liver disease	3 (1.6)	7 (0.7)	0.178
Bleeding diathesis	6 (3.3)	38 (3.6)	0.804
Smoking			**<0.001**
active or abstinence of <1 year	18 (9.8)	353 (33.7)	
abstinence of >1 year	58 (31.5)	284 (27.1)	
Excessive alcohol intake	9 (4.9)	40 (3.8)	0.493

Data are presented as frequency (N) and percentage (%) for categorical and as mean ± standard deviation for continuous variables unless specified otherwise. Due to rounding or missing data, totals may differ from 100%. Bold values denote statistical significance at the *p* ≤ 0.05 level. * This category includes the use of drug-eluting stents and drug-coated balloons. ^†^ Run-off could not be assessed in patients where the popliteal segment was treated; the reported frequencies and *p* value were calculated based on the number of patients with assessed run-off. PAD—peripheral arterial disease. ABI—ankle–brachial index. TIA—transient ischaemic attack.

**Table 2:** Prescribed treatment regimen for the group of patients treated with full-dose anticoagulant therapy and the group of patients treated with antiplatelet therapy.

Prescribed Treatment Regimen			
Anticoagulant Group N = 184		Antiplatelet Group N = 1047	
ACT only	93 (50.5)	ASA + clopidogrel	575 (54.9)
ACT + ASA	50 (27.2)	ASA	439 (41.9)
ACT + clopidogrel	14 (7.6)	clopidogrel	23 (2.2)
ACT + ASA + clopidogrel	27 (14.7)	other treatment regimens	10 (1.0)

Data are presented as frequency (N) and percentage (%). ACT—anticoagulant treatment. ASA—acetylsalicylic acid.

**Table 3:** Effectiveness and safety outcomes within the observed one-year period after revascularisation for the group of patients treated with full-dose anticoagulant therapy and the group of patients treated with antiplatelet therapy, before propensity score matching.

	Unmatched Anticoagulant Group N = 164	Unmatched Antiplatelet Group N = 929
**Adverse events included in the composite effectiveness outcome**	**71 (43.3)**	**228 (24.5)**
All-cause death	33 (20.1)	75 (8.1)
Exacerbation of PAD on the target limb	27 (16.5)	140 (15.1)
Major amputation of the target limb	11 (6.7)	13 (1.4)
**Adverse events included in the composite safety outcome**	**6 (3.6)**	**15 (1.6)**
Non-fatal major bleeding	5 (3.0)	13 (1.4)
Fatal bleeding	1 (0.6)	2 (0.2)

Data are presented as frequency (N) and percentage (%). PAD—peripheral arterial disease.

**Table 4:** Results of propensity score matching with the nearest-neighbour 1:5 algorithm with replacement for the group of patients treated with full-dose anticoagulant therapy and the group of patients treated with antiplatelet therapy.

	Unmatched Groups			Matched Groups		
	Anticoagulant Group N = 164	Antiplatelet Group N = 929	SMD	Anticoagulant Group N = 164	Antiplatelet Group N = 382	SMD
**Patient demographics**						
Age in years	76.5 ± 10.5	70.0 ± 10.2	0.624	76.5 ± 10.5	76.0 ± 9.4	0.049
Female sex	71 (43.3)	402 (43.3)	<0.001	71.0 (43.3)	157.9 (41.3)	0.040
**Markers of PAD severity and procedural characteristics**						
Fontaine classification grade			0.678			0.090
2b	67 (40.9)	675 (72.7)		67.0 (40.9)	140.2 (36.7)	
3	22 (13.4)	61 (6.6)		22.0 (13.4)	58.7 (15.4)	
4	75 (45.7)	193 (20.8)		75.0 (45.7)	183.1 (47.9)	
TASC II			0.235			0.074
A	20 (12.2)	166 (17.9)		20.0 (12.2)	42.9 (11.2)	
B	72 (43.9)	391 (42.1)		72.0 (43.9)	160.7 (42.1)	
C	60 (36.6)	338 (36.4)		60.0 (36.6)	143.9 (37.7)	
D	12 (7.3)	32 (3.4)		12.0 (7.3)	34.5 (9.0)	
not assessed	0 (0.0)	2 (0.2)		0.0 (0.0)	0.0 (0.0)	
Drug-coated device used *	5 (3.0)	31 (3.3)	0.016	5.0 (3.0)	15.8 (4.1)	0.059
Prior revascularisation			0.147			0.113
of the same segment	34 (20.7)	155 (16.7)		34.0 (20.7)	97.4 (25.5)	
of a different segment	18 (11.0)	140 (15.1)		18.0 (11.0)	40.5 (10.6)	
none	112 (68.3)	634 (68.2)		112.0 (68.3)	244.1 (63.9)	
Prior amputation			0.229			0.088
above the ankle	7 (4.3)	12 (1.3)		7.0 (4.3)	19.1 (5.0)	
below the ankle	14 (8.5)	49 (5.3)		14.0 (8.5)	41.5 (10.9)	
none	143 (87.2)	868 (93.4)		143.0 (87.2)	321.4 (84.1)	
Run-off			0.220			0.048
good	74 (45.1)	520 (56.0)		74.0 (45.1)	164.0 (42.9)	
poor	50 (30.5)	219 (23.6)		50.0 (30.5)	123.9 (32.4)	
not assessed	40 (24.4)	190 (20.5)		40.0 (24.4)	94.1 (24.6)	
**Cardiovascular risk factors and associated illnesses**						
Arterial hypertension	151 (92.1)	805 (86.7)	0.177	151.0 (92.1)	352.7 (92.3)	0.009
Dyslipidaemia, treated with statins	110 (67.1)	737 (79.3)	0.279	110.0 (67.1)	266.5 (69.8)	0.058
Diabetes mellitus	83 (50.6)	394 (42.4)	0.165	83.0 (50.6)	202.2 (52.9)	0.046
Ischaemic heart disease	48 (29.3)	205 (22.1)	0.165	48.0 (29.3)	122.1 (32.0)	0.058
Congestive heart failure	65 (39.6)	95 (10.2)	0.723	65.0 (39.6)	138.8 (36.3)	0.068
Prior stroke or TIA	40 (24.4)	104 (11.2)	0.350	40.0 (24.4)	86.2 (22.6)	0.043
Chronic kidney disease	71 (43.3)	234 (25.2)	0.389	71.0 (43.3)	171.0 (44.8)	0.029
Liver disease	3 (1.8)	6 (0.6)	0.107	3.0 (1.8)	5.6 (1.5)	0.029
Bleeding diathesis	6 (3.7)	34 (3.7)	<0.001	6.0 (3.7)	11.6 (3.0)	0.034
Smoking			0.659			0.050
active or abstinence <1 year	16 (9.8)	329 (35.4)		16.0 (9.8)	35.4 (9.3)	
abstinence >1 year	52 (31.7)	253 (27.2)		52.0 (31.7)	130 (34.0)	
non-smoker	96 (58.5)	347 (37.4)		96.0 (58.5)	216.6 (56.7)	
Excessive alcohol intake	8 (4.9)	35 (3.8)	0.055	8.0 (4.9)	20.0 (5.2)	0.017

Data are presented as frequency (N) and percentage (%) for categorical and as mean ± standard deviation for continuous variables. Due to replacement being enabled, frequencies after matching are shown with decimals, as statistical weights were considered. Totals may differ from 100% due to rounding. * This category includes the use of drug-eluting stents and drug-coated balloons. SMD—standardised mean difference. PAD—peripheral arterial disease. TIA—transient ischaemic attack.

## Data Availability

The data presented in this study are available on request from the corresponding author. The data are not publicly available due to privacy restrictions.

## Supplementary Materials

The following are available online at https://www.mdpi.com/article/10.3390/jcdd9070207/s1. Table S1: results of propensity score matching of all studied patients with the nearest-neighbour 1:5 algorithm with replacement for the group of patients treated with full-dose anticoagulant therapy and the group of patients treated with antiplatelet therapy; Figure S1: graphic presentation of the dispersion of SMD values for the group of patients treated with full-dose anticoagulant therapy and the group of patients treated with antiplatelet therapy, before and after matching with the nearest-neighbour 1:5 algorithm with replacement when considering all patients.
