# Supplementary material for: Clinical Outcomes after Endovascular Revascularisation of the Femoropopliteal Arterial Segment in Patients with Anticoagulant versus Antiplatelet Therapy: A Single-Centre Retrospective Cohort Study
Source: J Cardiovasc Dev Dis. 2022 Jun 30;9(7):207. doi: 10.3390/jcdd9070207 (PMC9319551; doi:10.3390/jcdd9070207)
Supplement: Supplementary file 1 [file jcdd-09-00207-s001.zip › jcdd-1766769-supplementary.pdf]

Supplementary Materials

This supplementary materials has been provided by the authors to give readers further information regarding their work.

Supplement to: Clinical Outcomes after Endovascular Revascularisation of the Femoropopliteal Arterial Segment in Patients with Anticoagulant versus Antiplatelet Therapy: A Single-Centre Retrospective Cohort Study

Subanalyses of the components of the composite effectiveness outcome

We performed two subanalyses, examining separately the components of the composite effectiveness outcome – all-cause death and MALE. The first was defined as death within one year after the revascularisation procedure, regardless of previous exacerbations of PAD symptoms or amputations. The second (MALE) was the exacerbation of PAD symptoms and previously unplanned major amputation of the treated limb due to vascular causes.

The propensity score matching (PSM) process was identical to the main analysis, meaning the same matched sample was used (Table 4). The relative treatment effect was assessed as described in the paper, using the same predictors, but with all-cause death or MALE as the outcome. We found no statistically significant difference in the occurrence of all-cause death and MALE between the ACT and APT group. The odds ratio (OR) for all-cause death with ACT versus APT was 0.56 (95% CI 0.24–1.29; p=0.176), while the OR for MALE with ACT versus APT was 1.61 (95% CI 0.77–3.37; p=0.204).

Sensitivity analysis using propensity score matching on all patients

We performed a sensitivity analysis using PSM and the same methodology as described in the paper. Unlike in the main analysis, the 138 patients who did not complete follow-ups were not excluded. We assumed they did not complete follow-ups due to clinical improvement, therefore assuming a best-case scenario where none of the adverse events in the composite effectiveness outcome (all-cause death, exacerbation of peripheral arterial disease symptoms, and previously unplanned major amputation of the treated limb due to vascular causes) occurred within one year after the procedure. In the observed time period, a major bleeding event occurred in one of the added 138 patients, which was taken into account in the analysis below.

The same methodology was used for PSM – optimal 1:1 matching, full matching without replacement, and nearest neighbour 1:5 matching with replacement were attempted. The most adequate balance was obtained by the last one.

The matched sample consisted of all patients in the anticoagulant (ACT) group and 440 patients in the antiplatelet (APT) group who matched best with the patients in the ACT group. When accounting for their statistical weights, the effective sample size of the APT group was 267.8 patients. The final standardised mean difference (SMD) values for all covariates before and after matching are listed in Table S1 and shown graphically in Figure S1.

Table S1. Results of propensity score matching of all studied patients with the nearest neighbour 1:5 algorithm with replacement for the group of patients treated with full-dose anticoagulant therapy and the group of patients treated with antiplatelet therapy.

	Unmatched groups – all patients			Matched groups		
	Anticoagulant group N=184	Antiplatelet group N=1,047	SMD	Anticoagulant group N=184	Antiplatelet group N=440	SMD
**Patient demographics**						
Age in years	76.4±10.5	70.5±10.3	0.572	76.4±10.5	76.7±9.3	0.033
Female sex	77 (41.8)	459 (43.8)	0.040	77.0 (41.8)	177.9 (40.4)	0.029
**Markers of PAD severity and procedural characteristics**						
Fontaine classification grade			0.626			0.072
2b	76 (41.3)	743 (71.0)		76.0 (41.3)	167.9 (38.2)	
3	25 (13.6)	69 (6.6)		25.0 (13.6)	58.3 (13.3)	
4	83 (45.1)	235 (22.4)		83.0 (45.1)	231.8 (48.6)	
TASC II			0.181			0.093
A	26 (14.1)	187 (17.9)		26.0 (14.1)	53.1 (12.1)	
B	81 (44.0)	443 (42.3)		81.0 (44.0)	186.5 (42.4)	
C	65 (35.3)	379 (36.2)		65.0 (35.3)	164.5 (37.4)	
D	12 (6.5)	36 (3.4)		12.0 (6.5)	35.9 (8.2)	
not assessed	0 (0.0)	2 (0.2)		0.0 (0.0)	0.0 (0.0)	
Drug-coated device used*	5 (2.7)	35 (3.3)	0.036	5.0 (2.7)	16.7 (3.8)	0.061
Prior revascularisation			0.142			0.113
of the same segment	36 (19.6)	169 (16.1)		36.0 (19.6)	101.4 (23.0)	
of a different segment	19 (10.3)	151 (14.4)		19.0 (10.3)	40.7 (9.2)	
none	129 (70.1)	727 (69.4)		129.0 (70.1)	298.0 (67.7)	
Prior amputation			0.217			0.069
above the ankle	9 (4.9)	20 (1.9)		9.0 (4.9)	26.8 (6.1)	
below the ankle	16 (8.7)	56 (5.3)		16.0 (8.7)	43.5 (9.9)	
none	159 (86.4)	971 (92.7)		159.0 (86.4)	369.7 (84.0)	
Run-off			0.173			0.090
good	85 (46.2)	573 (54.7)		85.0 (46.2)	184.6 (42.0)	
poor	55 (29.9)	256 (24.5)		55.0 (29.9)	146.8 (33.4)	
not assessed	44 (23.9)	218 (20.8)		44.0 (23.9)	108.6 (24.7)	
**Cardiovascular risk factors and associated illnesses**						
Arterial hypertension	170 (92.4)	909 (86.8)	0.183	170.0 (92.4)	409.9 (93.2)	0.029
Dyslipidemia	128 (69.6)	821 (78.4)	0.203	128.0 (69.6)	313.7 (71.3)	0.038
Diabetes mellitus	94 (51.1)	450 (43.0)	0.163	94.0 (51.1)	232.9 (52.9)	0.037
Ischaemic heart disease	58 (31.5)	229 (21.9)	0.219	58.0 (31.5)	146.8 (33.4)	0.039
Congestive heart failure	73 (39.7)	114 (10.9)	0.702	73.0 (39.7)	156.4 (35.5)	0.085
Prior stroke or TIA	44 (23.9)	120 (11.5)	0.331	44.0 (23.9)	97.6 (22.2)	0.041
Chronic kidney disease	77 (41.8)	267 (25.5)	0.351	77.0 (41.8)	203.7 (46.3)	0.090
Liver disease	3 (1.6)	7 (0.7)	0.090	3.0 (1.6)	4.3 (1.0)	0.058
Bleeding diathesis	6 (3.3)	38 (3.6)	0.020	6.0 (3.3)	19.1 (4.3)	0.057
Smoking			0.617			0.079
active or abstinence <1 year	18 (9.8)	353 (33.7)		18.0 (9.8)	33.5 (7.6)	
abstinence >1 year	58 (31.5)	284 (27.1)		58.0 (31.5)	145.9 (33.2)	
non-smoker	108 (58.7)	410 (39.2)		108.0 (58.7)	260.7 (59.2)	
Excessive alcohol intake	9 (4.9)	40 (3.8)	0.052	9.0 (4.9)	22.0 (5.0)	0.005

Data are presented as frequency (N) and percentage (%) for categorical and as mean ± standard deviation for continuous variables. Due to replacement being enabled, frequencies after matching are shown with decimals, as statistical weights were considered. Totals may differ from 100% due to rounding. *This category includes the use of drug-eluting stents and drug-coated balloons. SMD – standardised mean difference. PAD – peripheral arterial disease. TIA – transient ischaemic attack.

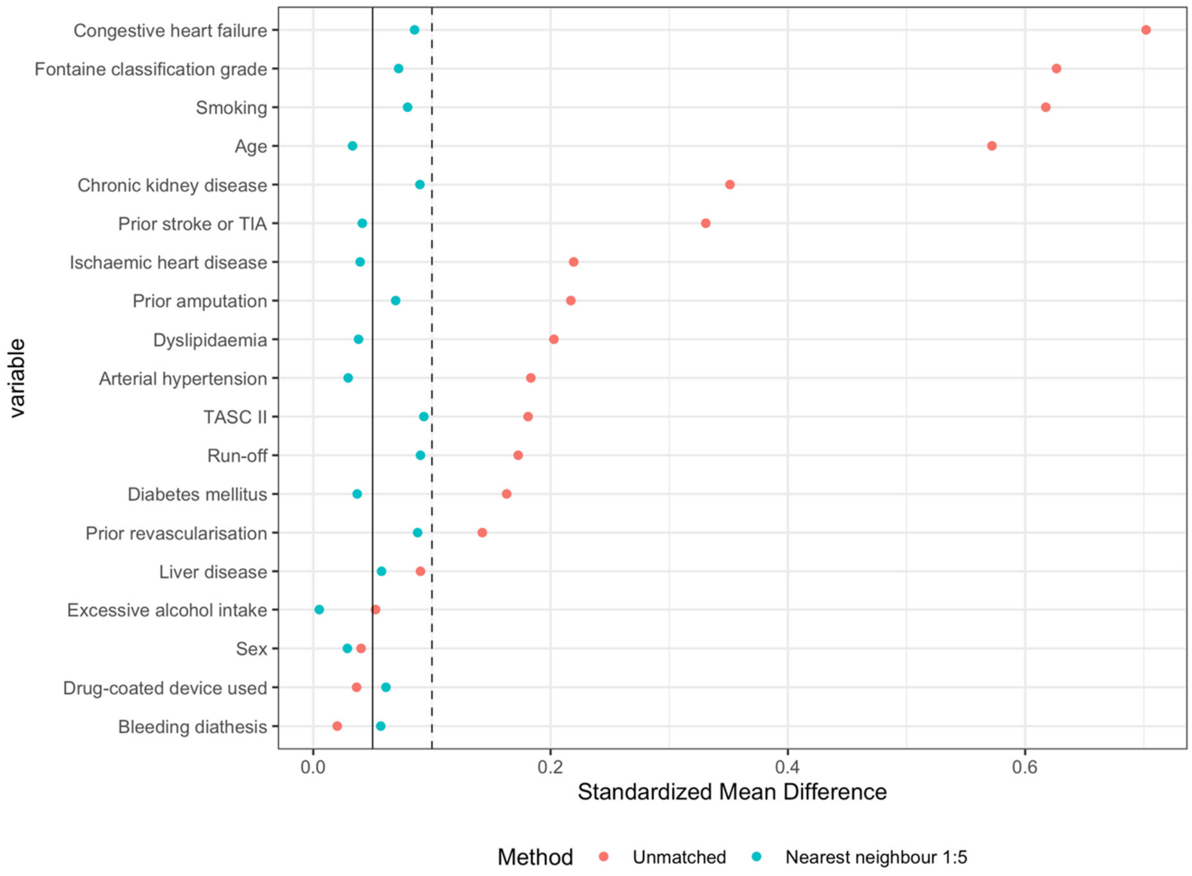

Figure S1. Graphic presentation of the dispersion of standardised mean difference (SMD) values for the group of patients treated with full-dose anticoagulant therapy and the group of patients treated with antiplatelet therapy, before and after matching with the nearest neighbour 1:5 algorithm with replacement when considering all patients.

After matching, we found no statistically significant difference in the effectiveness and safety of both treatment regimens. As in the paper, the logistic regression model with the treatment group, propensity scores and atrial fibrillation was used. The OR for the effectiveness outcome to occur with ACT versus APT was 1.32 (95% CI 0.74–2.37; p=0.346), while the OR for major bleeding to occur with ACT versus APT was 0.66 (95% CI 0.24–1.82; p=0.422).

Sensitivity analysis using multiple logistic regression on patients with both observed outcomes
A further analysis was performed on the unmatched sample of patients where both outcomes could be determined, meaning 164 patients in the ACT group and 929 patients in the APT group. Instead of using PSM to achieve treatment groups which were as comparable as possible, we used multiple logistic regression. As predictors we used atrial fibrillation and all variables used for PSM, excluding bleeding diathesis.

The OR for the effectiveness outcome to occur with ACT versus APT was 1.31 (95% CI 0.73–2.37; p=0.365), while the OR for major bleeding to occur with ACT versus APT was 0.57 (95% CI 0.10–3.37; p=0.536).
